# Modelling the effects of ephaptic coupling on selectivity and response patterns during artificial stimulation of peripheral nerves

**DOI:** 10.1371/journal.pcbi.1007826

**Published:** 2020-06-01

**Authors:** Miguel Capllonch-Juan, Francisco Sepulveda

**Affiliations:** BCI-NE group, CSEE, University of Essex, Colchester, United Kingdom; Eidgenossische Technische Hochschule Zurich, SWITZERLAND

## Abstract

Artificial electrical stimulation of peripheral nerves for sensory feedback restoration can greatly benefit from computational models for simulation-based neural implant design in order to reduce the trial-and-error approach usually taken, thus potentially significantly reducing research and development costs and time. To this end, we built a computational model of a peripheral nerve trunk in which the interstitial space between the fibers and the tissues was modelled using a resistor network, thus enabling distance-dependent ephaptic coupling between myelinated axons and between fascicles as well. We used the model to simulate a) the stimulation of a nerve trunk model with a cuff electrode, and b) the propagation of action potentials along the axons. Results were used to investigate the effect of ephaptic interactions on recruitment and selectivity stemming from artificial (i.e., neural implant) stimulation and on the relative timing between action potentials during propagation. Ephaptic coupling was found to increase the number of fibers that are activated by artificial stimulation, thus reducing the artificial currents required for axonal recruitment, and it was found to reduce and shift the range of optimal stimulation amplitudes for maximum inter-fascicular selectivity. During propagation, while fibers of similar diameters tended to lock their action potentials and reduce their conduction velocities, as expected from previous knowledge on bundles of identical axons, the presence of many other fibers of different diameters was found to make their interactions weaker and unstable.

## Author summary

The design of neural interfaces for artificial electrical stimulation in prostheses can greatly benefit from simulations using electrode-nerve interface models. Studies on electrical stimulation of nerves generally neglect the effects of ephaptic coupling (i.e., coupling between axon fibers and bundles due to their local electric fields) on axon responses to stimulation as ephaptic coupling is normally assumed to play no significant role in bundles of myelinated axons. However, studies have shown that ephaptic effects are non negligible in mammalian peripheral nerves. As such, we have built a resistor network model of a small peripheral nerve trunk for simulation of ephaptic interactions between axons under artificial electrical stimulation. Results show that ephaptic coupling clearly increases axon recruitment and, hence, influences selectivity. On the other hand, the ephaptic coupling effects during action potential propagation observed in this work are more complex than the known effects for ideal, homogeneous bundles. In this work, heterogeneity and variability of fiber diameters in a bundle were found to greatly diminish the strength of ephaptic interactions to the point where these interactions do not play a relevant role on action potential propagation.

## Introduction

Artificial sensory feedback is becoming a viable way to substantially improve the life quality of amputees [1, 2]. The task of providing it through neural interfaces saw a first success with [3], and later, sensory feedback was provided in a stable form in [4–6], where it helped human subjects to improve their performance at using bidirectional limb prostheses. In very recent years, new encoding strategies [2, 7, 8]—involving, mostly, electrode placement, resolution, and the modulation of amplitude and frequency in stimulating signals—have facilitated various forms of biomimetic—natural-like—sensory feedback [9, 10], which help subjects identifying grasped objects quicker [10], and improve task performance and embodiment [9]. However, further improvements are still needed towards full, naturalistic sensory restoration for complete rehabilitation of fine motor function and prosthesis embodiment.

The quality of artificial sensory feedback greatly depends on the quality of the interface between the artificial sensory device and the patient’s peripheral nervous system (PNS). Such neural interface needs to accurately target specific axons in order to elicit the desired sensations. For this, it is necessary to determine the optimal electrical stimulation patterns—in time, frequency, and space—, that maximise selectivity and accuracy during stimulation. Selectivity is the ability of a neural interface to target the desired axons for stimulation, while avoiding recruiting non-targeted axons. Optimising implant selectivity is not trivial and demands the use of in vivo experiments and/or computer simulations.

Computer models come, by definition, with limitations in accuracy compared to the results that could be obtained from experiments. On the other hand, they have the advantages of better affordability and usability as they can have quicker and cheaper setups. Simulations using computer models of electrode-PNS interfaces can be used to predict results from electrical stimulation and, ultimately, optimise electrode designs [11].

This field has successful precedents as in [12–14] or [15], whereby the ability of the models to predict the selectivity capabilities of the electrodes was experimentally validated [16]. These works use the innovative method of hybrid modelling, consisting of coupling Finite Element Methods (FEM) to solve the electric potential over a nerve, and neural compartmental modelling (using NEURON [17]) to solve resulting neural activity. Although these works use detailed geometrical representations of the nerves in their models, they rely purely on axon activation prediction to study selectivity and do not regard the effects that action potential (AP) propagation may have not only on the selectivity of the electrodes, but also on the frequency encoding of the signals that later reach the central nervous system. We believe that a more specific study is needed to assess the extent to which propagation can affect these variables.

In order to carry a detailed study of propagation, ephaptic interactions should be taken into account. Ephaptic interactions are normally disregarded for the case of myelinated axons due to the insulating properties of the myelin sheath, which makes the transverse component of the conductivity across the nerve much lower than the longitudinal component [18]. However, studies such as [19] and [20] provide insights about the existence and role of this effect during conduction on myelinated axons. Our hypothesis is that ephaptic interactions between axons in a peripheral nerve are likely to play a role in information processing through alteration of the relative timings between APs from different axons, and possibly, in the selectivity capabilities of the electrodes, in an similar way as in the olfactory nerve in mammals [21], and by mutual influence between axons during stimulation. Hence, including this type of coupling in the models might provide an improvement onto existing achievements in the predictions of electrodes’ fascicle selectivity and information encoding, which would in turn lead to more accurate and more naturalistic artificial sensory feedback in neural interfaces.

We have developed three-dimensional EMI-type [22] models of both realistic and ideal peripheral nerve trunks which use a Resistor Network (RN) in order to simulate stimulation and propagation with ephaptic interactions in a unique simulation, with the ultimate goal of using it towards making predictions of fascicle targeting selectivity, frequency encoding, and overall electrode performance, in order to optimise electrode designs. The significance of this work lies in being the first work, to the best of our knowledge, that studies Ephaptic Coupling (EC) for a nerve model with realistic details and which deviates from restrictive assumptions such as the Mean-Field (MF) model or more regular geometries [19–21, 23], and therefore intends to elucidate the relevance of EC in more realistic conditions. Furthermore, we present a complete and self-consistent EMI model, specific for models of nerves and bundles of cylindrical axons—models of mere bundles of axons or fibers can be distinguished in this work from nerve models by the absence of other elements, such as perineurial membranes separating fascicles—which uses an existing geometrical tessellation technique to model the nearest-neighbour electrical connections between fibers and tissues.

In summary, the main novelty of this work is the study of EC:

- for nerves and bundles of randomly-located myelinated mammal peripheral axons with varying diameters, following both uniform and natural-like distributions,
- that departs from MF assumptions and takes the inter-axonal distances into account through a RN,
- that, for this purpose, presents a method to quantify nearest-neighbour electrical connections for any distribution of axons and tissues across the nerve,
- in scenarios where the nerve models are stimulated by cuff electrodes.

## Results

### Field generated by the electrode

**Fig 1:**
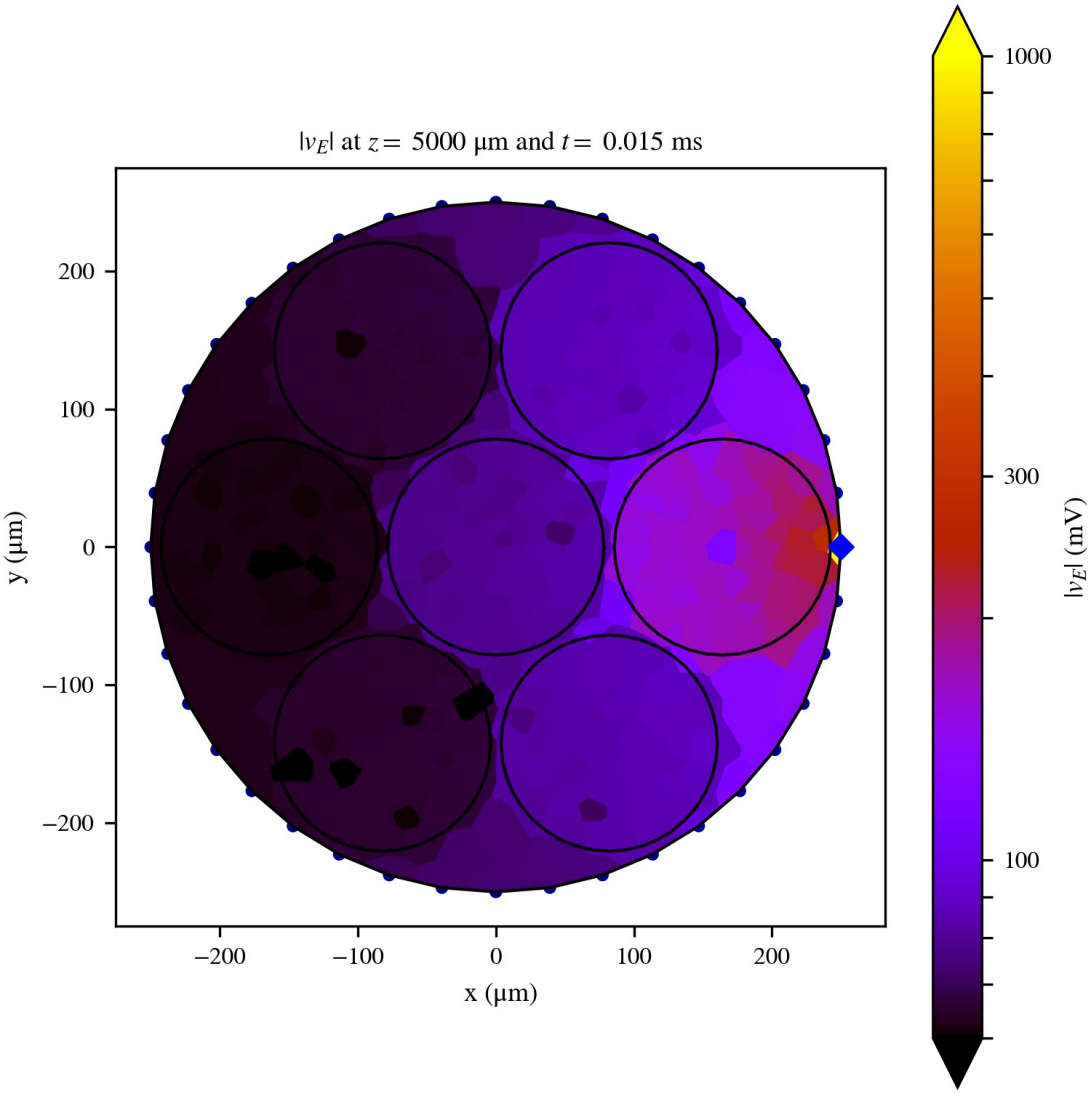
Cross-sectional slice of the extracellular field generated by the electrode. Cross-sectional slice of the extracellular field generated by the electrode over the model Nerve 1 at the middle of its length (*z* = 5000 *μ*m), where the stimulation pad (blue diamond) is situated, and at the time step following the onset of the stimulating pulse. The RN assumes the field is constant over the surface of each tessellation polygon. The contours of the nerve and the fascicles are indicated with a black solid line for better identification. Axons are not shown in this figure. Although the maximum value of |*v*_*E*_|, situated at the active site, is 2413.62 mV, the colorbar was cut at 1000 mV in order to facilitate the visualisation of the spatial details of the field.

In this subsection, we represent the extracellular potential field, *v*_*E*_, generated by the pulses exerted by one active pad on a cuff electrode on a nerve model. For this and the following subsection, we used a 1 cm long nerve (model named Nerve 1 in Methods) surrounded on part of its length by a stimulating cuff electrode that provided one square stimulating pulse. The cuff model was centered at the middle of the nerve’s length. The 0° pad (blue diamond on Fig 1) injected a square pulse with a duration of 200 *μ*s and an intensity of −3 *μ*A. The fiber diameters were randomly chosen following a distribution based on [24], although the diameters were bounded between 3 and 20 *μ*m. No fibers thinner than 3 *μ*m were taken into account, since low diameter fibers have short internodal lengths and would increase the RN resolution, along with the simulation’s computational cost. Considering that the fields obtained here are used in the stimulation studies in the next subsection, it is important to remark that, in order to save computational resources, the RN was connected only in the region under the cuffs. This was considered as a safe assumption since the fields far from the stimulation point were too small to play a relevant role during stimulation. The rest of the nerve’s length was left in order to avoid the effects of sealed-end boundary conditions of the axons.

**Fig 2:**
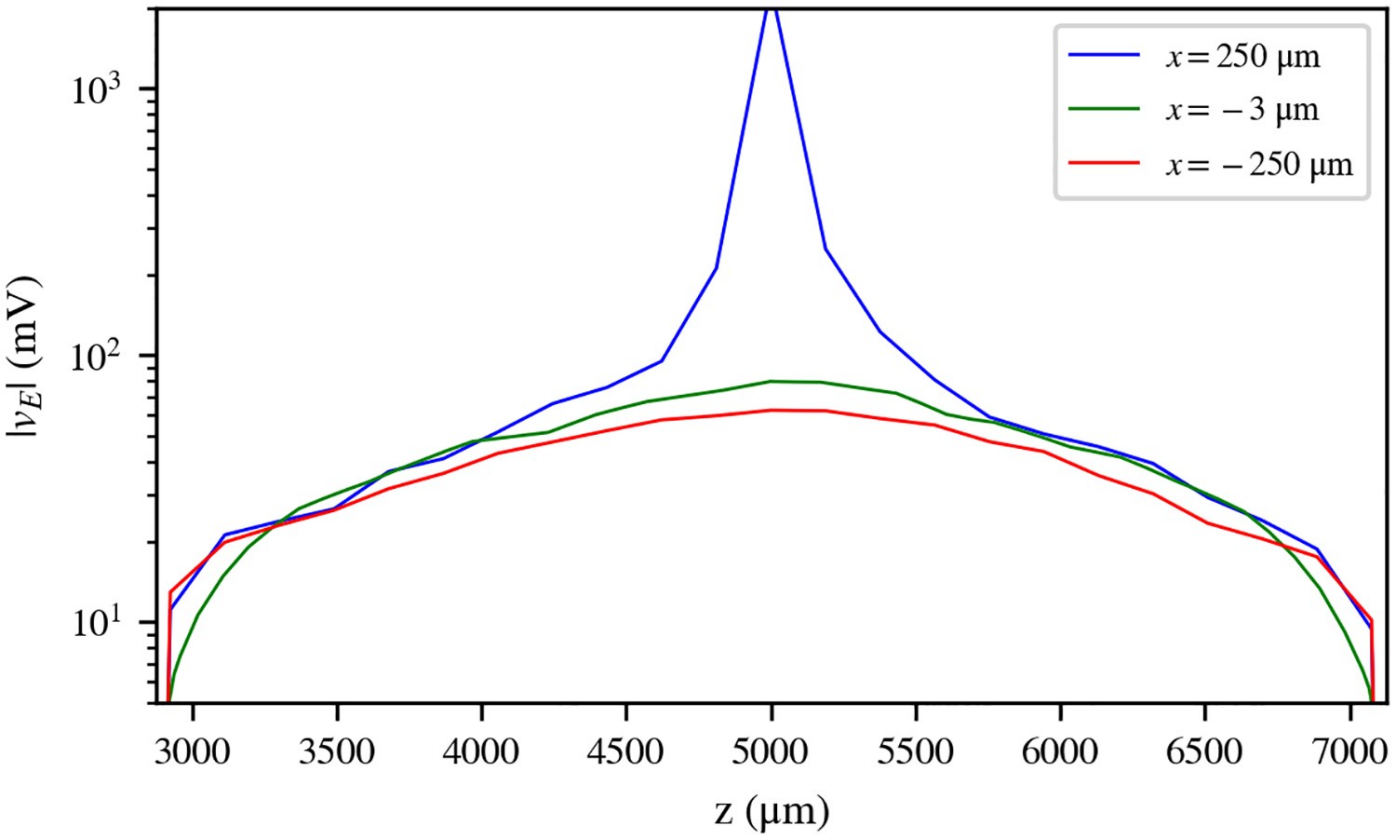
Longitudinal profile of the extracellular field generated by the electrode. Longitudinal profile (*z*-axis) of the extracellular field (absolute value, logarithmic scale) generated by the electrode over the model Nerve 1, along the length of the cuff electrode, at three different points on the *x*-*y* plane: the position of the active pad (*x* = 250 *μ*m, blue), the position of the central-most axon in the nerve (*x* = −3 *μ*m, green), and the farthest point from the active pad (*x* = −250 *μ*m, red). All three points are located at *y* = 0 *μ*m.

A cross-sectional view of the absolute value of the field *v*_*E*_ over the nerve can be seen in Fig 1, and three samples of its longitudinal profile (*z*-axis) can be seen in Fig 2. The field, which is negative across the entire domain, has a minimum value of −2413.62 mV at the location of the active pad, but its absolute value is lower than 1000 mV over the rest of the domain. The field can be seen to decrease with the distance from the active pad both in Fig 1 and in Fig 2. At the ends of the cuff, the field is effectively zero (Fig 2).

In simulations where the presence of the axons is merely accounted for by the anisotropy of the endoneurium’s resistivity tensor, a smooth dependence of |*v*_*E*_| with distance across the *x*-*y* plane from the active pad should be expected. However, in this simulation, axons are explicitly included in the RN. The resulting field (Fig 1) presents deviations from such a smooth dependence, at points where |*v*_*E*_| is generally low. This is due to the conductive axoplasm of the axons, which lowers the impedance to ground on their locations.

### Effects of ephaptic coupling on axon recruitment and selectivity

In order to study the effects of EC on axon recruitment and selectivity during stimulation, we tested the differences in stimulation results from simulations with and without EC. For this, we used the model Nerve 1 under the same conditions as the previous subsection. Two sets of simulations were run for this study: one including EC (labeled as SEC; results in Fig 1 for a pulse of −3 *μ*A) and one not including it (SNOEC). SEC simulations were run by modelling the nerve as a RN. SNOEC simulations were prepared in the following way: The axon models are the same as in SEC. However, there is no RN interconnecting the axons, and therefore, no explicit modelling of any extracellular tissue or device. In order to model stimulation, the extracellular fields along all the axons in SEC were captured at the time step following the start of the stimulating pulse, and then used in SNOEC as the extracellular stimulating field on the axons.

In order to quantify the effects of stimulation, we measured the axon recruitment in response to the stimulating pulses. The presence of APs on each fiber was detected when the transmembrane potential of the fiber (*v*_*m*_) reached 15 mV. This AP detection method was used throughout this study. We ran pairs of simulations {SEC, SNOEC} for current pulse amplitudes ranging from −0.2 to −4 *μ*A, with steps of −0.2 *μ*A.

**Fig 3:**
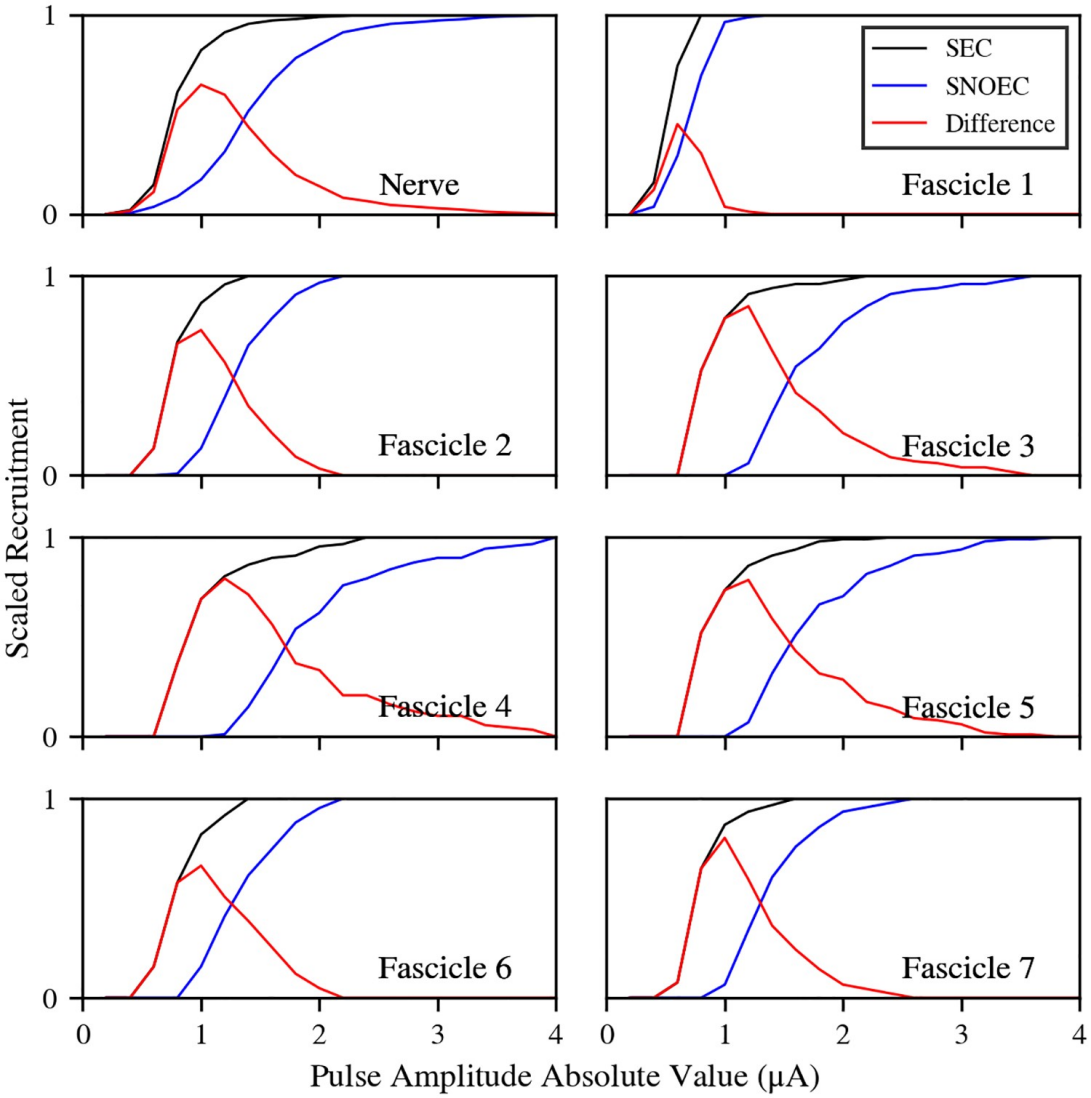
Recruitment curves. Scaled recruitment curves for all the fascicles and the whole nerve. Black lines correspond to SEC and blue lines correspond to SNOEC simulations. Red lines show the difference between the two. The horizontal axis indicates the pulse amplitudes exerted on the electrode’s active pad. Pulses are always negative in the simulations, but they have been represented as absolute values in this figure for clarity.

**Fig 4:**
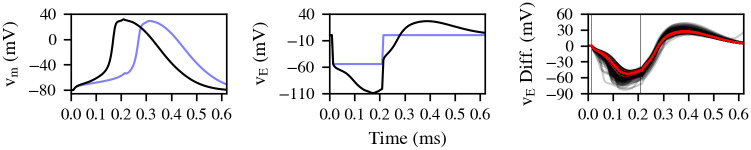
Ephaptic field. For one particular axon, randomly chosen as an example, central panel shows the time evolution of the extracellular potential (*v*_*E*_) on the node of Ranvier lying closer to the electrode’s active pad for both simulations (blue for SNOEC, black for SEC), and left panel shows the time evolution of the transmembrane potential (*v*_*m*_, same location and legend). Note in this panel how the EC produces an AP earlier than in SNOEC. Right panel: Time evolution of the endogenous fields ($v_{E}^{SEC}-v_{E}^{SNOEC}$) for all the axons (thin black lines) on the nodes lying closer to the active pad. Red lines indicate the mean of these fields (averaged for each time step, middle thick line) with their standard deviation (thin lines). The two black vertical lines indicate the start and finish of the pulse.

The method used for the stimulating fields in SNOEC ensures the axons are stimulated with the same field coming from the electrode in SNOEC and SEC. However, results vary substantially between both cases (Fig 3). The recruitment in SEC is, for all fascicles and pulse amplitudes, higher than in SNOEC, and it is also triggered for smaller currents. Recruitment ratios are only equal between SEC and SNOEC in trivial cases: when recruitment is zero and when it is saturated (i.e., the maximum number of axons in a fascicle has been recruited) in both simulations. This is due to the endogenously generated field (or ephaptic field), which adds up to the artificial field from the electrode and generates an increased depth of the total stimulating field over all axons (see Fig 4, where the ephaptic field and membrane voltage are represented for a random axon under a stimulating pulse with amplitude −2 *μ*A). The ephaptic field activates axons by pushing them over their activation thresholds, where the electrode fields alone are not enough. Hence, the ephaptic field effectively reduces the threshold current for axon recruitment. In the simulation with a stimulating pulse amplitude of −2 *μ*A, this ephaptic field is deeper than −50 mV on average (right panel) although it reaches depths in the range between −60 and −80 mV for some axons. There are no axons for which this field is positive throughout the duration of the stimulating pulse. It does, however, become positive after the pulse, likely due to the refractory periods of the axons.

This model contains 658 axons, most of which are firing APs at similar times in SEC for strong enough stimulating pulses. From a MF model perspective, this means that the individual contribution to the ephaptic field from each axon might be in the order of, at least, 10 *μ*V. In cases where an electrode is set to selectively target a group of axons, the collective influence of these on the ephaptic field may be lower, and therefore, the effects on axon recruitment may be lower as well. Nevertheless, we can tell that the magnitude of the effect of EC on the axons response is big enough to be taken into account unless working with much smaller groups.

**Fig 5:**
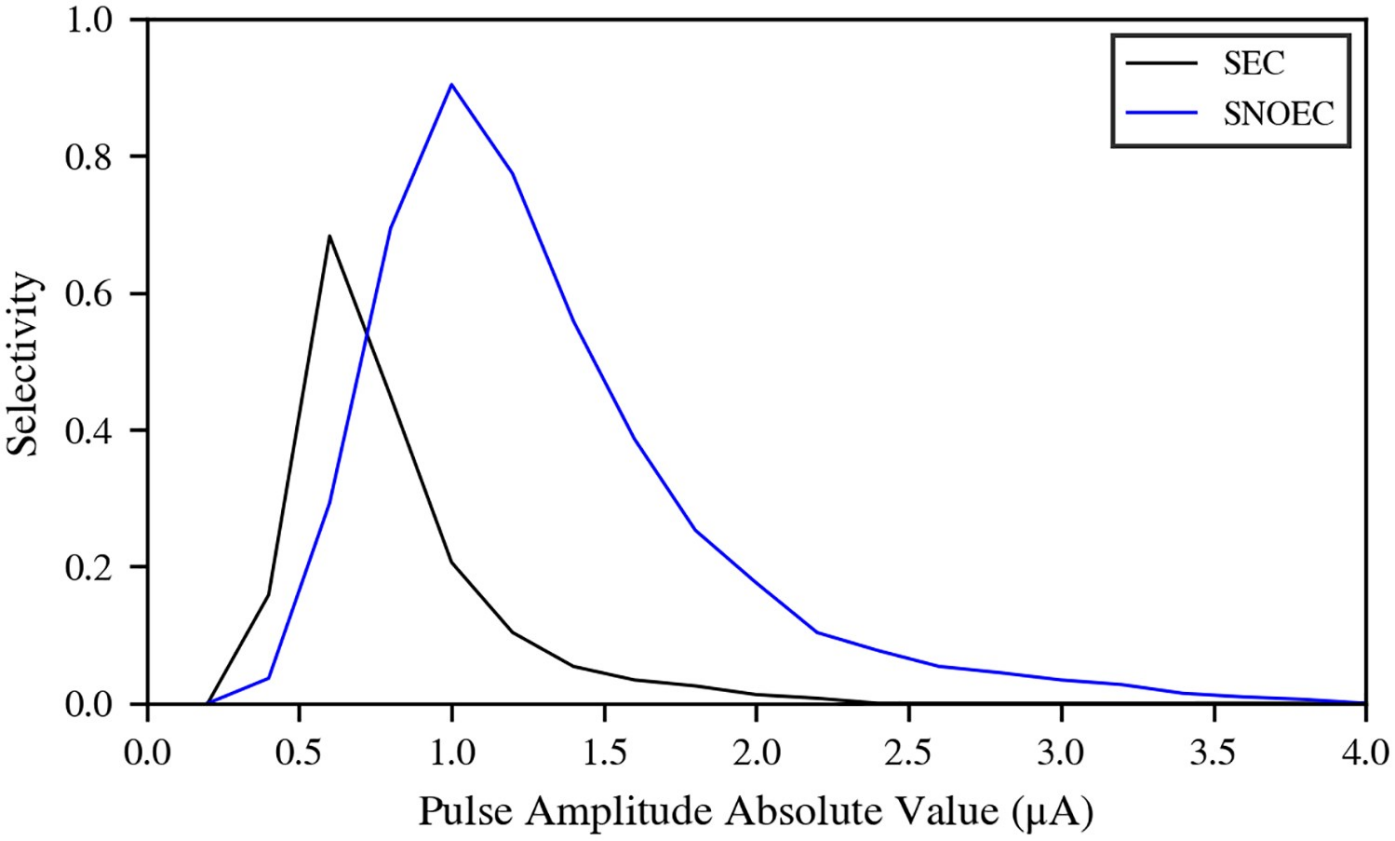
Selectivity for Fascicle 1. Selectivity for Fascicle 1 for the various pulse amplitudes in use.

The position of the active pad with respect to Fascicle 1 was assumed to be the optimal for maximising the selectivity for this fascicle. We studied the variation of the selectivity for Fascicle 1 with the presence of EC. We used the inter-fascicular selectivity provided by [15], and calculated its value for the whole range of stimulating pulse amplitudes. Results (Fig 5) indicate that EC, in the case of Fascicle 1, has the effect of narrowing the range of pulse amplitudes for which the selectivity is optimal by approximately 0.5 *μ*A, and shifting the peak of the selectivity also by approximately 0.5 *μ*A. Also, the maximum selectivity that can be reached is lower than in SNOEC. This can be understood thanks to the increase in axon recruitment in all other fascicles for pulse amplitudes from −0.6 *μ*A and stronger. Recruitment in Fascicle 1 is always higher than in the other fascicles thanks to its proximity to the active pad, and it reaches its maximum recruitment sooner. Therefore, selectivity for Fascicle 1, while using only the current active pad, cannot be negative. The possible effects of EC on the selectivity of other fascicles, however, may be different, since their optimal selectivity configurations vary.

### Effects of ephaptic coupling on propagation

We intended to study the effects that EC may have on propagation of APs. For this, we used the same approach: we ran a pair of simulations, SEC and SNOEC, on the same model, using the same stimulation protocol, and their results were compared.

Propagation with EC needs to be studied along a longer model than Nerve 1, and for a longer period of time. Increasing the length of Nerve 1 highly increases the computational demands of simulations, so we instead used a thinner mono-fascicular nerve model: Bundle 1 (see Methods), which is 6 cm long and has a diameter of 100 *μ*m. No perineurial tissue was taken into account. In order to increase the effects of EC, the epineurial walls of the bundle were given the same resistivity as the cuffs, thus providing a virtual quasi-isolation from the surrounding saline bath. The bundle’s ends were not covered by this isolating surface, so the tissues were in contact with the paths to ground on those two surfaces.

Bundle 1 contains 39 axons whose fiber diameters follow a continuous and uniform distribution, in the range from 9 *μ*m to 10.9 *μ*m, and in steps of 0.05 *μ*m. This range was chosen so that the conduction velocities (CVs) did not vary drastically and thus to facilitate the possibility of signal locking between fibers of similar diameters.

An intracellular current injection was given to all axons on their first node of Ranvier, consisting of one square pulse of 10 nA at *t* = 0.01 ms with a duration of 10 *μ*s.

**Fig 6:**
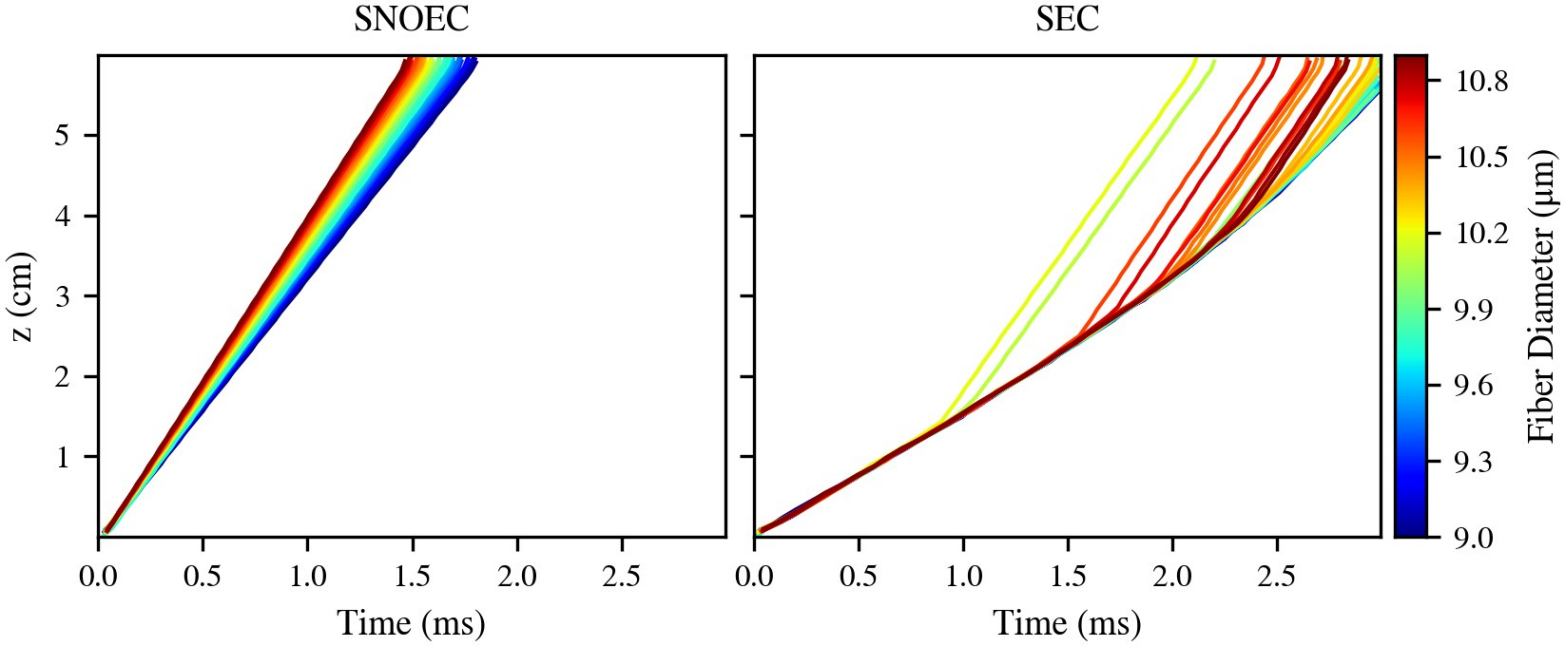
Propagation: Action potential trajectories. Trajectories of the axons on the *t*-*z* space for SNOEC and SEC. Each trajectory is coloured according to its corresponding fiber’s diameter. These results correspond to Bundle 1.

Results (Fig 6) show the presence of an effective lock of the APs in SEC during the first 0.5 to 1 ms of the simulation. However, it is apparent that this lock is unstable: After around 1 ms, APs tend to detach from the main group along time and increase their CVs. The first APs in detaching do not belong to the higher diameter fibers, but rather, to mid-to-high diameter fibers. These are then followed by higher diameter fibers. As a first hypothesis to explain this observation, this could be due to the loss of a bond between the higher and lower diameter fibers when the mid-to-high diameter fibers depart. However, the causes of this generalised detachment of trajectories from the main AP lock can be numerous and complex. The weakness—or instability—of the EC between fibers of different diameters could be explained by the differences in the CVs they tend to have in the absence of EC, which would act against locking their APs. The observation that these detachments occur after a certain distance along the *z*-axis suggests the presence of factors that trigger the separation of APs when certain conditions are met. One of this is, potentially, the variation along the *z*-axis of the alignment between nodes of Ranvier of different axons, which would modify the strength of their EC.

**Fig 7:**
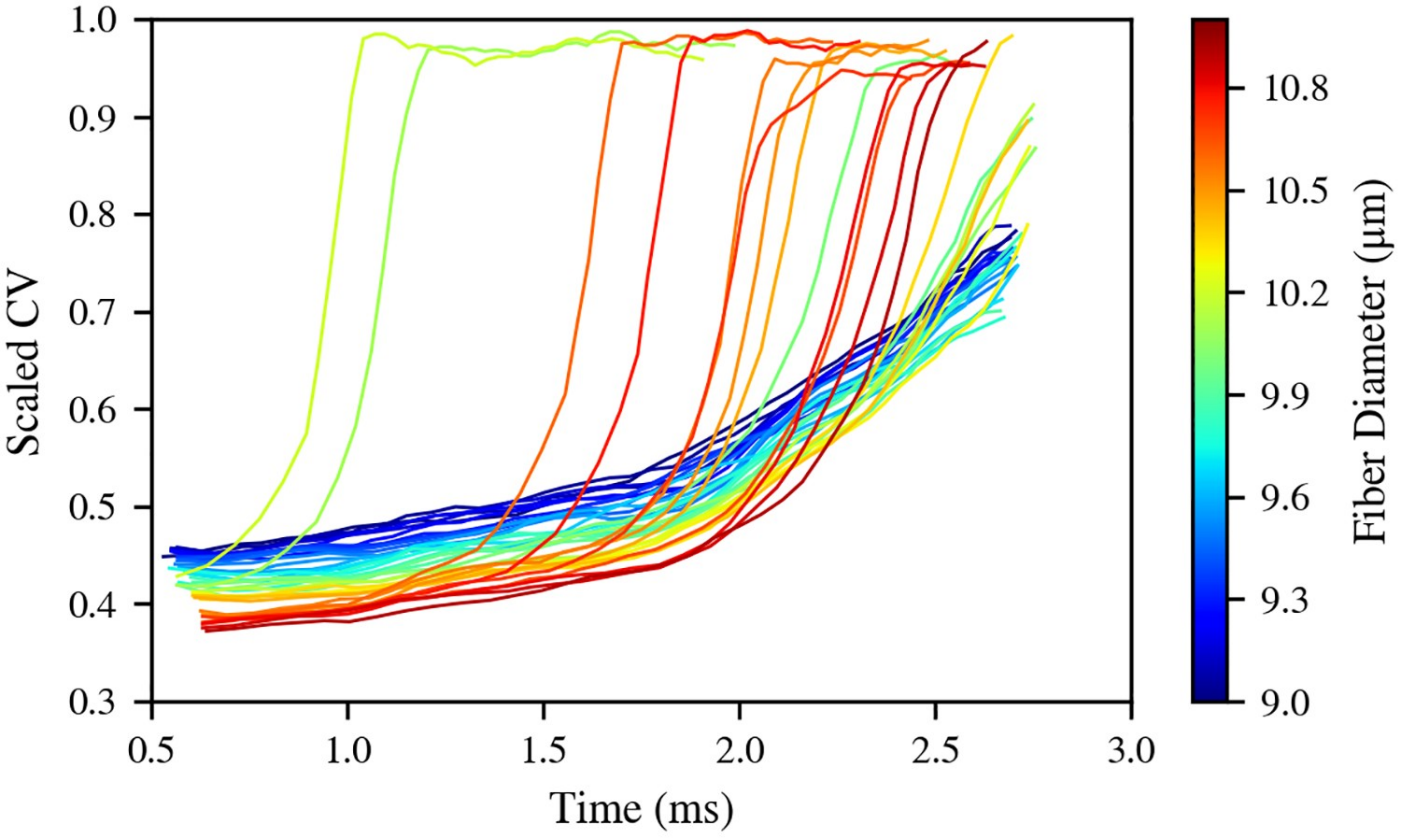
Propagation: Conduction velocities. CVs of the fibers in the simulation SEC, scaled over their respective values in SNOEC, which are stationary. CVs are obtained from a linear regression on the (*t*, *z*) points of the trajectories, using a window of 11 nodes or Ranvier, so the curves do not span the whole simulation. Error margins are not shown in order to aid a clearer visualisation. These data correspond to Bundle 1.

The CVs of the fibers can be directly related with the presence of their APs in or outside the AP lock. APs that separate from the group quickly reach the CVs they have in the absence of EC (Fig 7). At the beginning of the simulation, when all the APs form a locked group, they all have CVs of less than half of the values they have in SNOEC. These CVs in the lock, however, gradually increase along time as APs separate from the lock.

**Fig 8:**
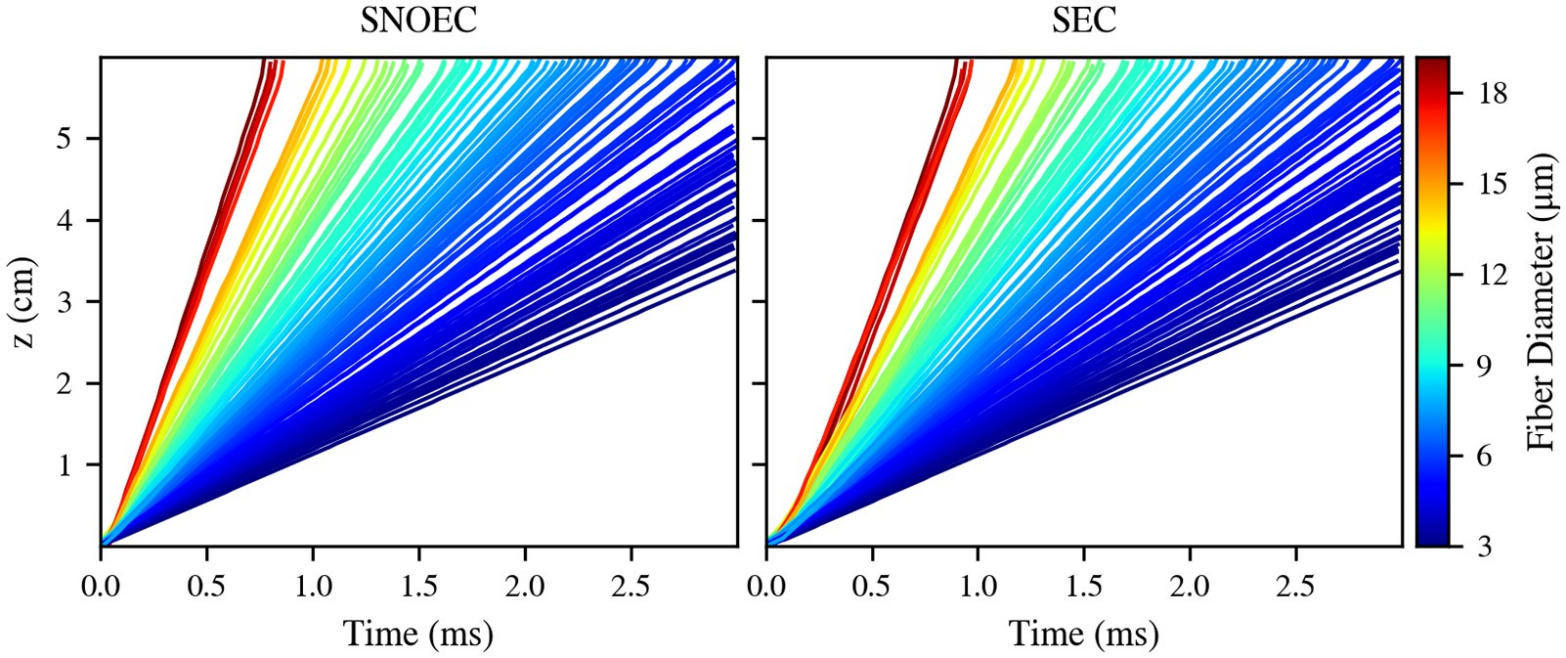
Propagation in a bundle with a natural fiber diameter distribution: Action potential trajectories. Trajectories of the axons on the *t*-*z* space. Each trajectory is coloured according to its corresponding fiber’s diameter. These results correspond to Bundle 2.

The same simulation sets were run for Bundle 2, although the stimulating pulse was 20 nA. Bundle 2 follows a natural diameter distribution (starting from 3 *μ*m), and the bundle diameter is larger than in Bundle 1 in order to facilitate the presence of more axons, and hence, a smoother diameter variability within the model. Results (Fig 8) indicate much weaker or nearly nonexistent AP lockings in SEC. This is in contrast with the apparent, although unstable and temporary, locking seen for Bundle 1. This is probably due to the wide range of different diameters in Bundle 2. However, a general slowdown of the CVs is still present in SEC. From these results, it is apparent that the strength of the effects of EC on the propagation of APs is highly dependent on the diameter variability between the fibers in a bundle.

### Dependence of ephaptic interactions with distance

We ran two simulations in which we stimulated one random axon in each with an internal current injection and observed the responses of the other (unstimulated, meaning they were not artificially stimulated) axons transmembrane potentials. We compared these responses to the distances from the artificially unstimulated axons to the artificially stimulated axon.

In this study, we used two models in order to study different scenarios, which differ in the presence of fascicles separated by perineurium:

- Bundle 3 is a 3 cm long, 250 *μ*m diameter, mono-fascicular nerve filled with 20 *μ*m diameter fibers. This model has a larger diameter than Bundle 1 because we wanted to obtain a characterisation of the strength of EC across a wider cross-sectional distance. As is the case of Bundle 1 and Bundle 2, no perineurium is considered. Also, in this and the model below, the epineurial walls of the models were strongly isolated from the saline bath.
- Nerve 2 uses the same epineurial and perineurial profile as Nerve 1—it has the same contours for the nerve and the fascicles cross-section—but it is filled exclusively with 20 *μ*m diameter fibers, as Bundle 3, and is also 3 cm long.

**Fig 9:**
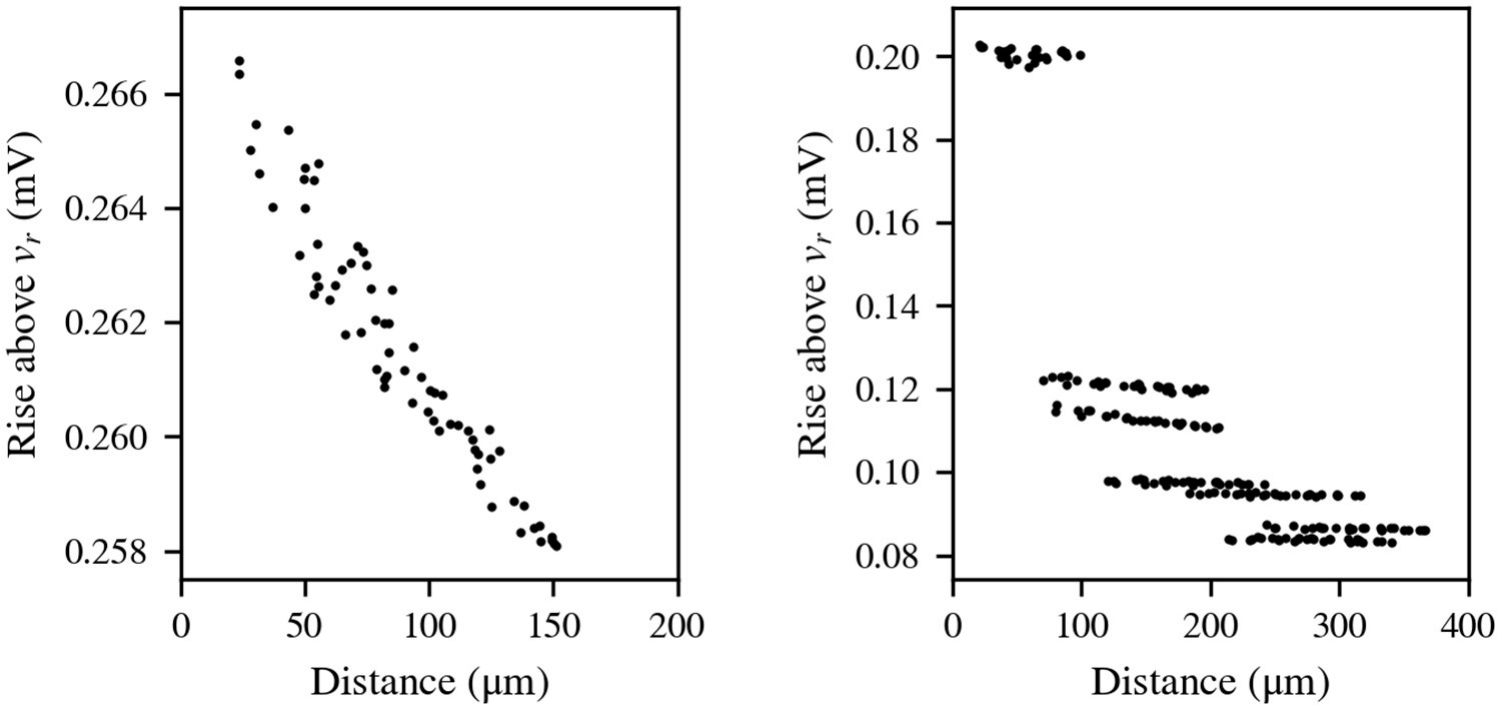
Strength of ephaptic coupling with inter-axonal distance. Maximum variation of *v*_*m*_ above *v*_*r*_ (resting potential, −80 mV) along the unstimulated axons, represented against the distance to the stimulated axon. Left: Bundle 3; right: Nerve 2, which contains seven fascicles separated by a perineurium, same as Nerve 1.

Results for Bundle 3 are shown in Fig 9 (left), and results for Nerve 2 are shown in Fig 9 (right). The responses of the unstimulated axons in Bundle 3 follow a clear decreasing trend with the distance from the stimulated axon. The irregularities can be attributed to the limitations of the RN at modelling three-dimensional space and to inter-axonal ephaptic interactions between unstimulated axons. Nevertheless, the total change of the responses along 150 *μ*m of distance does not vary much above 8 *μ*V. This suggests the acceptability of the application of a MF assumption in cases like this model, since variations on *v*_*m*_ of this order of magnitude would not imply big differences in the results from MF and distance-based EC simulations. It is important to bear in mind, however, that this order of magnitude in the unstimulated axons responses is due to the activity of one stimulated axon only. The combined effect of more axons carrying APs would increase it.

The responses in Nerve 2 are larger, near 0.2 mV inside the fascicle where the stimulated axon is, and approximately between 0.08 and 0.12 mV for the other fascicles. This is in rough agreement with the order of magnitude estimated in the second subsection of the Results if we have in mind that the nerve’s length affects this magnitude (a longer length increases the resistance to the saline bath or ground, which increases this magnitude). Axons belonging to different fascicles are easy to identify in Fig 9 (right), since the isolation provided by the perineurium makes the response of all axons inside each fascicle similar between them but notably different to the responses in other fascicles. The order of magnitude of these results could mean that the responses would be in the order of several mV should there be more stimulated axons, as seen in Fig 4. However, the intrafascicular variations are, at least, one order of magnitude lower. This would support a local MF choice for each fascicle. However, this choice would be incompatible with modelling inter-fascicle ephaptic interactions or fields from extracellular electrodes.

These observations, especially when considering the activity of many axons taking place in simulations, support the importance of choosing a distance-based model.

## Discussion

The model framework developed in this study permits simulating the stimulation and propagation on a peripheral nerve trunk in a single run. The framework introduces a new method to build nearest-neighbour electrical interactions between fibers which builds up a whole electrical network for the nerve. This network simulates the fields coming from electrodes and from the fibers, thus enabling the integrated simulation of EC.

This model has the advantage of being able to simulate the interactions between fibers and electrodes as well as with all other fibers in a nerve, where the nerve may have any reasonable shape, contain any number of fascicles separated by perineurial membranes and randomly located fibers of various diameters. However, running this with a reasonable level of computational efficiency has only been possible, so far, by accepting a series of assumptions and limitations:

- Axons are cylindrical and use a 1D cable equation. The effects of the transverse components of polarisation around the membranes are not regarded. Although these effects have been found to play no major role in myelinated axons when studying stimulation [25], no study has been done on their influence on ephaptic interactions of two very nearby cells. Only [26] provide simulation results which could provide clues on this, yet it is not their main focus.
- Axons are straight. However, tortuosity could affect EC by modifying the nearest-neighbour connections of the axons along the *z*-axis.
- Electrical currents through space are only modelled along the *z*-axis and on the *x*-*y* plane. A FEM scheme could simulate these currents more accurately.
- No capacitive properties have been regarded for any extracellular tissues.
- Unmyelinated axons are not regarded in this model. Although of low relevance for our purposes, a more complete model should take them into account. Further work should assess the effects of EC under the conditions studied here (i.e., stimulation with electrodes and propagation in heterogeneous bundles) for unmyelinated axons, and quantify ephaptic interactions between unmyelinated and myelinated fibers.
- The largest nerve model we have used in this work has a diameter of 500 *μ*m, and contains fascicles with a diameter of 156.67 *μ*m. These numbers are smaller than the known physiological ranges for human limb peripheral nerves where stimulation is typically studied [27, 28]. Also, fiber packing ratios are generally lower than physiological values. Therefore, the models used in this work contain less axons than real nerves. The computation time of the RN is highly sensitive to increasing the number of axons in the model. Hence, using physiologically more plausible numbers of axons would have been unattainable.

Further improvements on some of the limitations of this model can be carried out in further work. These range from increasing the variety of axon models in use, to including capacitive properties of tissues, and adding tortuosity. The latter could be achieved by dividing the nerve’s length in layers, each layer having its particular arrangement of fiber positions according to their tortuosity and hence, having its particular power diagram.

Computational cost is generally a drawback for simulations with this model. Calculations over a RN are expensive and this limits the size and resolution that the model can have in order to get reasonable simulation time investments. Parallelisation of the RN could not be done, to the best of our efforts, without compromising numerical stability. This resolution limitation also compromises the accuracy of the results of simulations with EC, since small changes in the RN resolution or arrangement have large effects on EC.

Laguerre tessellations are used for building nearest-neighbour connections between fibers. This method is used for the study of granular structures, like polycrystals and foams [29–31], whose field of application is strikingly different from the applications of this work. Yet, it proves to be a convenient method for modelling these connections, since it provides a general tool which serves any possible packing of cylindrical fibers. Prior to this work, no similar approach has been found for this purpose. Point or line-source approximations [32] can be used for this. However, even their adaptations to anisotropic media neglect the complexities the nerve may have outside each individual fiber, which can turn into an inaccurate modelling when these complexities are important. Also, using the equations from [32] in our case of mutual EC between many fibers may lead to numerical instability, as seen in [33]. Furthermore, no study has been found so far using any distance-based approach for a similar type of nerve model.

This has allowed us to simulate stimulation and propagation in a somewhat realistic nerve model. From the numerical simulations presented here, we have found that EC lowers stimulation current thresholds and, overall, drives an increased axon recruitment (compared to simulations that neglect EC) during stimulation with a cuff electrode. The thresholds are lowered by an amount of the order of 100 nA for Fascicles 1, 2, 6 and 7, and of 1 *μ*A for Fascicles 3, 4, and 5. The increase in recruitment has a maximum of 64.9% for the whole nerve, and it is above 60% for all fascicles, except for Fascicle 1 (Fascicle 2: 72.9%; Fascicle 3: 84.8%; Fascicle 4: 79.3%; Fascicle 5: 78.6%; Fascicle 6: 66.3%; and Fascicle 7: 80.2%). Fascicle 1 has a maximum recruitment increase of 45.1%. For all the former fascicles, this maximum seems to be centered around a stimulating pulse of −1 *μ*A, and around −0.6 *μ*A for Fascicle 1, which closely correspond to their respective threshold currents in SNOEC. Therefore, these high peak levels in recruitment difference are mostly resolved from the fact that EC lowers the stimulation thresholds by nearly 1 *μ*A. So, for pulses near the peak, EC drives APs in a large number of axons that lie under their thresholds in simulations without EC. The lower threshold reduction and recruitment difference in Fascicle 1 compared to the others can be explained from the relative value of the ephaptic field with respect to the electrode field: While the ephaptic field has an order of magnitude of 10 mV, and may vary within a range of the same order over the whole nerve (as seen for a pulse of −2 *μ*A in Fig 3), the electrode field has, in general, also an order of magnitude of 10 mV in all fascicles, except Fascicle 1, where it is one order of magnitude stronger (see Figs 1 and 2). Therefore, axons located in distant fascicles can be more sensitive to the ephaptic field. Recruitment difference decreases for stronger (i.e., more negative) pulses than −1 *μ*A for Fascicle 1 and −0.6 *μ*A for other fascicles, even when stimulation has not reached its maximum in SEC, because axons start activating in SNOEC.

The inter-fascicular selectivity for Fascicle 1 was studied for a range of pulse amplitudes in order to determine how EC affects selectivity for the fascicle lying nearest to the active pad. It has been found that EC has the effects of 1) narrowing the range of pulse amplitudes resulting in high selectivity by approximately 0.5 *μ*A, 2) shifting the peak of the selectivity toward smaller pulse amplitudes in absolute value by approximately 0.5 *μ*A, and 3) reducing the maximum attainable selectivity from 0.9 to 0.68.

We have observed how axons interact between them during stimulation, and although the strength of the individual influence from one axon is generally weak, their collective interactions are determinant to whether axons lying close to their thresholds fire an AP or not. We used a configuration where axons of different diameters are uniformly spread across the nerve’s cross-section. This is representative of proximal sections of nerves. However, more distal sections present clustering of fiber types and diameters. This is known to affect the spread of activation thresholds within a fascicle [34], so further studies would be necessary to assess the validity of these findings in such configurations. The possibility of AP firing due to EC during propagation has not been studied in this work. In the study of the dependence of EC with distance, the observed rise in *v*_*m*_ of axons was due to the activity of only one neighbouring axon. It is inferred, from the orders of magnitude under consideration, and from the observed ephaptic fields in the stimulation study, that the simultaneous activity of many more axons could drive unstimulated axons to fire APs. Although studying this possibility is outside the scope of this present work, it is proposed as further work.

By following these considerations, EC should be taken into account in simulations of axon recruitment with electrodes, but if it is to be neglected in favour of lower computational costs, it should at least be held in mind that neglecting it may lead to certain inaccuracies in the results. Ideally, such a study lacking explicitly modelled EC could consider these effects by applying a modifying function to recruitment numbers after a simulation. The results in this work suggest that amplitudes of stimulating pulses necessary for axon recruitment in experimental studies and practical applications should be generally lower than as obtained from models lacking EC.

We have observed how, in this work, certain already existing findings [19–21] about the effects of EC on few fibers during propagation—CV reduction and AP locking—also apply for bundles with more numerous and heterogeneous fibers. However, these effects are strongly conditioned by similarity between fibers and compromised by heterogeneity to the point of losing their validity when assumptions of homogeneous fibers are not used. It is apparent that fiber diameter variability in a bundle greatly influences the effects of EC on AP propagation. This implies that the effects of EC on propagation might be weak, and even irrelevant, in proximal sections of nerves, where fiber diameters are homogeneously distributed, but they could be stronger, and forming effective lockings, in more distal regions, where fibers may be clustered by size.

The results of this work also have assessed the validity of choosing a MF model: although physically not accurate and unsuitable for studies involving extracellular electrodes, it can be justified for others, especially for small mono-fascicular nerves or locally within fascicles.

In summary, a detailed computer model of a peripheral nerve trunk has been developed, which involves the implicit coupling of intra- and extracellular electrical activity in a single simulation. It conveniently uses NEURON with a Python framework that handles all the geometrical methods and wraps the whole model. Specific experimental data for validation would be desirable. However, the model succeeds in behaving within physiologically expected ranges. We hope that this new method provided here brings researchers to use it further in order to study more complex cases of ephaptic interactions, and that the results from this study serve to add more knowledge on the effects of EC in bundles of fibers with different sizes, eventually to determine the extent to which modelling EC for studying sensory feedback is necessary.

## Methods

The fundamental assumptions on which the model is based, the axon models in use and a detailed description of the procedures used to model the nerve’s tissues are provided here.

### Main assumptions and limitations

The model relies on several assumptions to simplify the implementation and computational cost while still keeping an acceptable level of accuracy:

1. Only two types of axon models are used: the double-cable models of McIntyre, Richardson and Grill (MRG) [35] for motor fibers, and Gaines & al. [36] for sensory fibers. No unmyelinated or other types of myelinated axons are considered.
2. Axons are straight, with no tortuosity (i.e., with no bends, undulations, or tapering) along their length.
3. All axons are parallel to each other.
4. Following the two above assumptions, the cross-section of the nerve’s anatomy is constant along its length.
5. All extracellular tissues are purely ohmic.
6. The volumes of the epineurium and endoneurium are regarded as part of a three-dimensional RN.
7. The endoneurium was modelled as an isotropic tissue, since using its anisotropic tensor from [37] would imply an over-representation of the axons.
8. The perineurium is regarded as a surface with a nominal thickness influencing the values of the resistances that cross it.
9. The nearest-neighbour electrical connections model defines inter-axonal connections only across the *x*-*y* plane, and inter-compartmental connections along the *z*-axis. This is a limitation with respect to FEM schemes, which can model currents flowing in any direction.
10. The RN is computationally expensive. A very large number of axons in the model can greatly increase the simulation time to days. Therefore, although the typical diameters of human limb peripheral nerves where stimulation is studied are in the order of several mm [27, 28], we used smaller nerve models and axon bundles (see Table 1 for more details). Also, fiber packing ratios and axon numbers were kept low.

**Table 1:** Geometrical and electrical properties of the models.

Model	Diameter (*μ*m)	Number of axons	Fiber packing ratio	Intracellular to extracellular areas ratio	Length (cm)
Nerve 1	500	658			1
Fascicle 1 (Nerve 1)	156.67	82	0.282	0.205	1
Fascicle 2 (Nerve 1)	156.67	118	0.350	0.267	1
Fascicle 3 (Nerve 1)	156.67	99	0.344	0.269	1
Fascicle 4 (Nerve 1)	156.67	87	0.290	0.212	1
Fascicle 5 (Nerve 1)	156.67	98	0.330	0.251	1
Fascicle 6 (Nerve 1)	156.67	83	0.293	0.225	1
Fascicle 7 (Nerve 1)	156.67	91	0.283	0.193	1
Bundle 1	100	39	0.398	0.304	6
Bundle 2	150	110	0.347	0.267	6
Bundle 3	250	69	0.450	0.606	3
Nerve 2	500	192			3
Fascicle 1 (Nerve 2)	156.67	26	0.429	0.555	3
Fascicle 2 (Nerve 2)	156.67	28	0.462	0.634	3
Fascicle 3 (Nerve 2)	156.67	28	0.462	0.634	3
Fascicle 4 (Nerve 2)	156.67	27	0.445	0.593	3
Fascicle 5 (Nerve 2)	156.67	29	0.478	0.677	3
Fascicle 6 (Nerve 2)	156.67	27	0.445	0.593	3
Fascicle 7 (Nerve 2)	156.67	27	0.445	0.593	3

### Axon and nerve models

**Fig 10:**
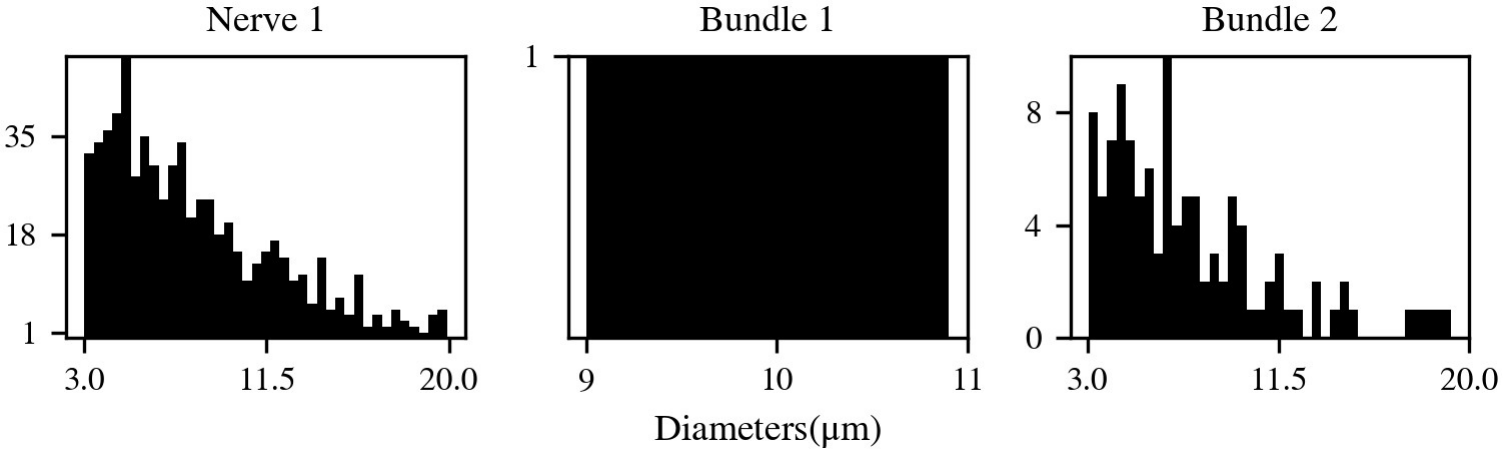
Histograms for fiber diameters. Histograms for fiber diameters of the nerve and bundle models used in this study, except for models without diameter variability (Bundle 4 and Nerve 2). Horizontal axes indicate diameter values in *μ*m and vertical axes indicate the number of axons for each bin of the histograms. Note that although all histograms have the same number of bins (39), they do not necessarily share any horizontal or vertical axes. The corresponding model names are indicated on the top of each histogram.

A number of different models were used in this work in order to run the different studies (see Table 1 for a detailed list of these models and Fig 10 for their fiber diameter distributions). Model named Nerve 1 in this work uses both motor and sensory fiber models, with a proportion of 15% motor and 85% sensory fibers [36]. All other models use, exclusively, motor fibers. In all cases, we used a temperature of 37°C. Unmyelinated fibers are known to be generally more numerous than myelinated fibers in peripheral nerves [38, 39]. Also, EC in unmyelinated axons can be relatively strong. However, their use in our model implied a high computational cost due to the higher spatial resolution that they require. Furthermore, they are outside the scope of this study as our focus is on the often neglected EC between myelinated fibers. Therefore, unmyelinated fibers were not included in the models presented here.

In Nerve 1 and Bundle 2, the fiber diameters were randomly assigned following a distribution according to the results in [24], although they were bound between 3 *μ*m and 20 *μ*m (smaller diameter fibers were excluded due to their fine spatial discretisation requirements, which led to higher computational costs). Therefore, the nodes of Ranvier of the different axons were not necessarily aligned. The different properties of the fiber morphology that depend on the diameter—internodal length, morphology of the myelin attachment (MYSA) and paranodal (FLUT) regions and number of myelin layers, were fitted to a linear regression each, using the values from [35]. Variables whose linear regressions yielded negative values were fitted to a quadratic curve, as done in [40].

The implementation of the axon membrane models was made in the NEURON simulation environment [17].

For Nerve 1 and Nerve 2, we used a nerve model as a cylindrical body with seven cylindrical fascicles of equal diameter, inspired in the five-fascicle model from [12]. In all models, the fascicles were filled with axons using a simple circle packing algorithm designed for this purpose.

The algorithm consisted of one iterative process for each fascicle where, in each iteration, a random diameter value *D*_*k*_ was chosen from the aforementioned distribution for a circle (a fiber, indexed with *k*). For each circle, a loop for positioning trials was then run. On each trial, a random position for the center of the circle was chosen inside the fascicle (more specifically, inside a circle having a diameter *D*_*F*_ − *D*_*k*_, being *D*_*F*_ the diameter of the fascicle, in order to avoid intersection of the circle with the fascicle’s membrane). If the circle at the position had no intersections or contacts with any other circle that had been placed previously in the fascicle, the position was assigned to it and a new random circle was chosen. The algorithm stopped when a circle could not be placed at a suitable position after 10, 000 trials. For this process, a minimum allowed distance between axons was chosen to be 1 *μ*m (which was taken into account at each contact check), so no two axons could be closer to each other than that. Table 1 summarises the fiber packing results for the different models used in this work. For this, values for each model are shown of the fiber packing ratio, defined as *A*_*F*_/*A*_*T*_, and of the total intracellular to extracellular areas ratio, defined as *A*_*ax*_/*A*_*E*_, where *A*_*F*_ is the sum of the cross-sectional areas of the fibers, including their myelin sheaths, *A*_*ax*_ is the sum of their cross-sectional intracellular areas, *A*_*E*_ is the total cross-sectional extracellular area of the model, and *A*_*T*_ is its total cross-sectional area. This algorithm can fill fascicles of any shape with fibers.

The algorithm used here yields fiber packing ratios which are generally lower than the typical values in nerves (see, for instance, [41] for measured values in human spinal cord). However, these lower ratios prevented us from having a very high number of axons, which would increase the computational cost of the simulations.

Three different extracellular tissues were considered in the model (Table 2): The epineurium was used for the whole extrafascicular space inside the nerve, the endoneurium was used to account for all the intrafascicular spaces where axons were embedded, or interstitial spaces, and the perineurium was regarded as a surface layer that electrically separated the fascicles from the epineurium. Nevertheless, the epineurium and the endoneurium were given the same electrical properties for the following reasons, respectively: The epineurium was considered to be isotropic as in [12, 15]. The endoneurium’s resistivity taken from the literature [37] is considered to be anisotropic because it accounts for the longitudinal disposition of the axons. In this RN, however, axons are explicitly represented by implementing their membranes and intracellular resistances as part of the RN. Using the known value from [37] for the longitudinal component of the endoneurium’s resistivity, $\rho_{En}^{L}=175$ Ω ⋅ cm, is then not suitable for this model, since that would imply an over-representation of the intracellular resistances. Hence, given the lack of knowledge about the value of $\rho_{En}^{L}$, we made the conservative assumption of considering the endoneurium as an isotropic tissue, and used its transverse component of the resistivity, $\rho_{En}^{T}$, as the value for its longitudinal component.

**Table 2:** Parameters used for the RN.

Symbol	Value	Source	Description
*ρ*_*ax*_	70 Ω ⋅ cm	[35]	Axoplasmic resistivity.
$\rho_{En}^{L}$	1211 Ω ⋅ cm	[37]	Longitudinal (*z*-axis) component of the resistivity of the endoneurium. See main text to understand the discrepancy with the anisotropic tensor from [37].
$\rho_{En}^{T}$	1211 Ω ⋅ cm	[37]	Transverse (*x*-*y* plane) component of the resistivity of the endoneurium.
$\rho_{Ep}^{L}$	1211 Ω ⋅ cm	[12, 15]	Longitudinal component of the resistivity of the epineurium.
$\rho_{Ep}^{T}$	1211 Ω ⋅ cm	[12, 15]	Transverse component of the resistivity of the epineurium.
$\rho_{P}^{T}$	1.136 ⋅ 10^5^ Ω ⋅ cm	[11]	Transverse (and only) component of the resistivity of the perineurium (value for 37° see reference).
*ρ*_*I*_	10^9^ Ω ⋅ cm	[12]	Resistivity of the insulator.
*ρ*_*S*_	50 Ω ⋅ cm	[12]	Resistivity of the saline bath.
Δ_*P*_	4.7 ⋅ 10^−4^ cm	[42]	Thickness of the perineurium (3% of the fascicle diameter in Nerve 1; see Table 1).
Δ_*I*_	2.4 ⋅ 10^−2^ cm	[12]	Thickness of the insulating cuff.
Δ_*S*_	0.85 cm		Thickness of the saline bath in the cylindrical container (in the absence of cuffs).
Δ_*C*_	2.2 cm	[12]	Cylindrical container’s diameter.
*D*_*N*_	0.5 cm		Diameter of the nerve.
*n*_*H*_	36		Number of points in the triangulation hull (or number of NAELC on the nerve’s membrane).

### Resistor network model

The whole extracellular volume of the nerve is modelled with a RN which uses [43] as the basic model of the extracellular medium between two cables. Ours is an adaptation from such model that suits any number of myelinated axons and also volumes in the nerve that contain no axons.

#### Extracellular space connecting two neighbouring axons

The model from [43] consists of two parallel core-conductor (unmyelinated) axons linked by a grid of resistors. Each axon is coupled to its parallel (longitudinal) extracellular cable through its membrane compartments, and the two longitudinal extracellular cables are linked to each other by transverse resistors *R*_*T*_—perpendicular to the axons, at each compartment’s position. Each longitudinal extracellular cable is a series of resistors with value *R*_*L*_ located one at each compartment.

**Fig 11:**
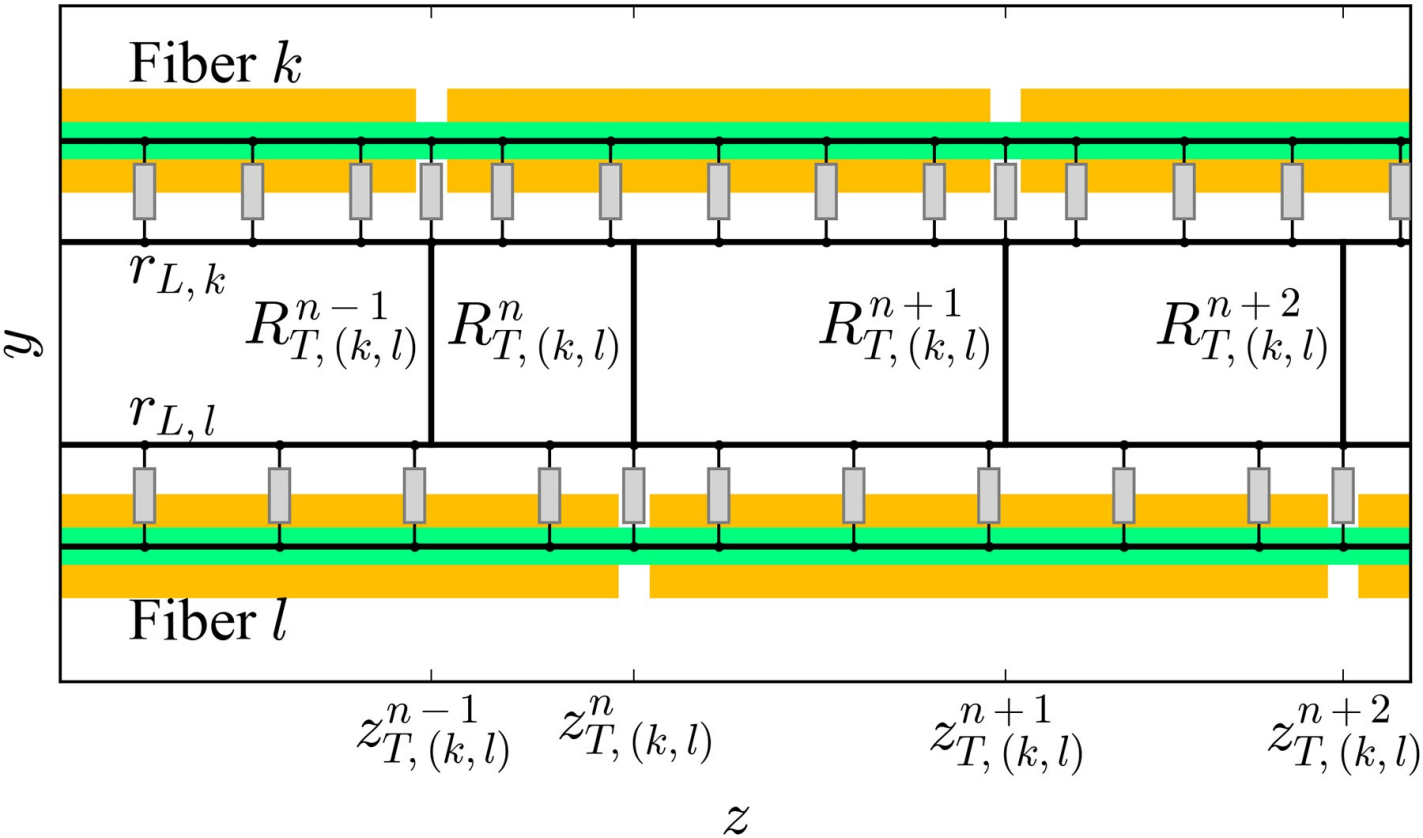
Resistor network connecting two myelinated fibers ephaptically. Example of RN connecting two myelinated fibers ephaptically. Conceptual (not to scale) representations of two myelinated fibers are shown as axons (green) wrapped by the myelin sheaths (dark yellow). Thick black line segments represent purely resistive connections. Grey boxes represent membrane compartments, either nodal or internodal (in which case, they also include the myelin sheath in series). The periaxonal space of the double-cable model is not shown in this figure for simplicity, but it is important to hold in mind that it is present in the models. The *y*-axis has been used on the ordinate axis in this figure for simplicity, but given our model, this can be any direction co-planar with the *x*-*y* plane.

Two important adaptations are needed in case the model includes myelinated axons with different internodal lengths and therefore, with misaligned nodes of Ranvier (Fig 11): First, the extracellular cables of the fibers are continuous resistive cables along which transverse resistors can be connected at any location. Second, there are two options for how to connect the transverse resistors: The first one is to locate them at regular intervals along the *z*-axis. The second one consists of connecting them at the locations of the nodes of Ranvier of both axons (this is the case shown in Fig 11). In this case, the set of transverse resistor locations along the *z*-axis between any two fibers *k* and *l* is *Z*_*T*,(*k*,*l*)_, which is the union of the sets of positions of the nodes of the two axons:

$$Z_{T,(k,l)}=Z_{k}\cup Z_{l},$$ (1)

and therefore, it contains *M*_*k*,*l*_ elements (which means there are *M*_*k*,*l*_ transverse resistors between the two axons; see Table 3 for a list of all the variables used here), i.e., the sum of the number of nodes of Ranvier of the two axons minus the number of pairs of nodes which share the same location on the *z*-axis (because such case, obviously, means there is only one resistor for two nodes):

$$M_{k,l}=N_{k}+N_{l}-\sum_{i=1}^{N_{k}}\sum_{j=i}^{N_{l}}\delta (z_{NR,i}-z_{NR,j}),$$ (2)

where *z*_*NR*,*i*_ (*z*_*NR*,*j*_) is the position of the *i*-th (*j*-th) node of Ranvier of fiber *k* (*l*).

The length along the *z*-axis of one transverse resistor *n* is given by:

$$c_{k,l}^{n}=\frac{z_{T,(k,l)}^{n+1}-z_{T,(k,l)}^{n-1}}{2},$$ (3)

being $z_{T,(k,l)}^{n}$ a member of *Z*_*T*,(*k*,*l*)_:

$$z_{T,(k,l)}^{n}\in Z_{T,(k,l)}\forall n|n\in \left[1,M_{k,l}\right]$$ (4)

**Table 3:** Variables used for the RN.

Symbol	Units	Description
*a*_*k*,*i*_	None	Fraction of cross-sectional area of tissue of type *i* present in polygon *k*.
$b_{k,l}^{i}$	None	Distance crossed through a tissue of type *i* by the transverse resistor between *k* and *l* as a fraction of the total distance between the membranes of *k* and *l*.
*A*_*P*,*k*_	cm^2^	Cross-sectional area of polygon *k*.
*A*_*E*,*k*_	cm^2^	Extracellular cross-sectional area inside polygon *k*.
*D*_*k*_	cm	Diameter of fiber *k* (zero for NAELC).
$c_{k,l}^{n}$	cm	Length (along the *z*-axis) of the transverse resistor number *n* between cables *k* and *l*.
*d*_*C*,(*k*,*l*)_	cm	Distance between the centers of fibers *k* and *l*.
*s*_*k*,*l*_	cm	Length of the segment in common between polygons *k* and *l*.
$\rho_{k}^{Lu,L}$	Ω ⋅ cm	Longitudinal component of the lumped resistivity for polygon *k*.
$\rho_{k,l}^{Lu,T}$	Ω ⋅ cm	Transverse component of the lumped resistivity between cables *k* and *l*
*r*_*L*,*k*_	Ω/cm	Resistance per unit length of the extracellular cable *k*.
$R_{T,(k,l)}^{n}$	Ω	Value of the transverse resistor *n* between cables *k* and *l*
*R*_*G*_	Ω ⋅ cm	Resistance to ground from a point on the nerve’s membrane per unit length.

**Fig 12:**
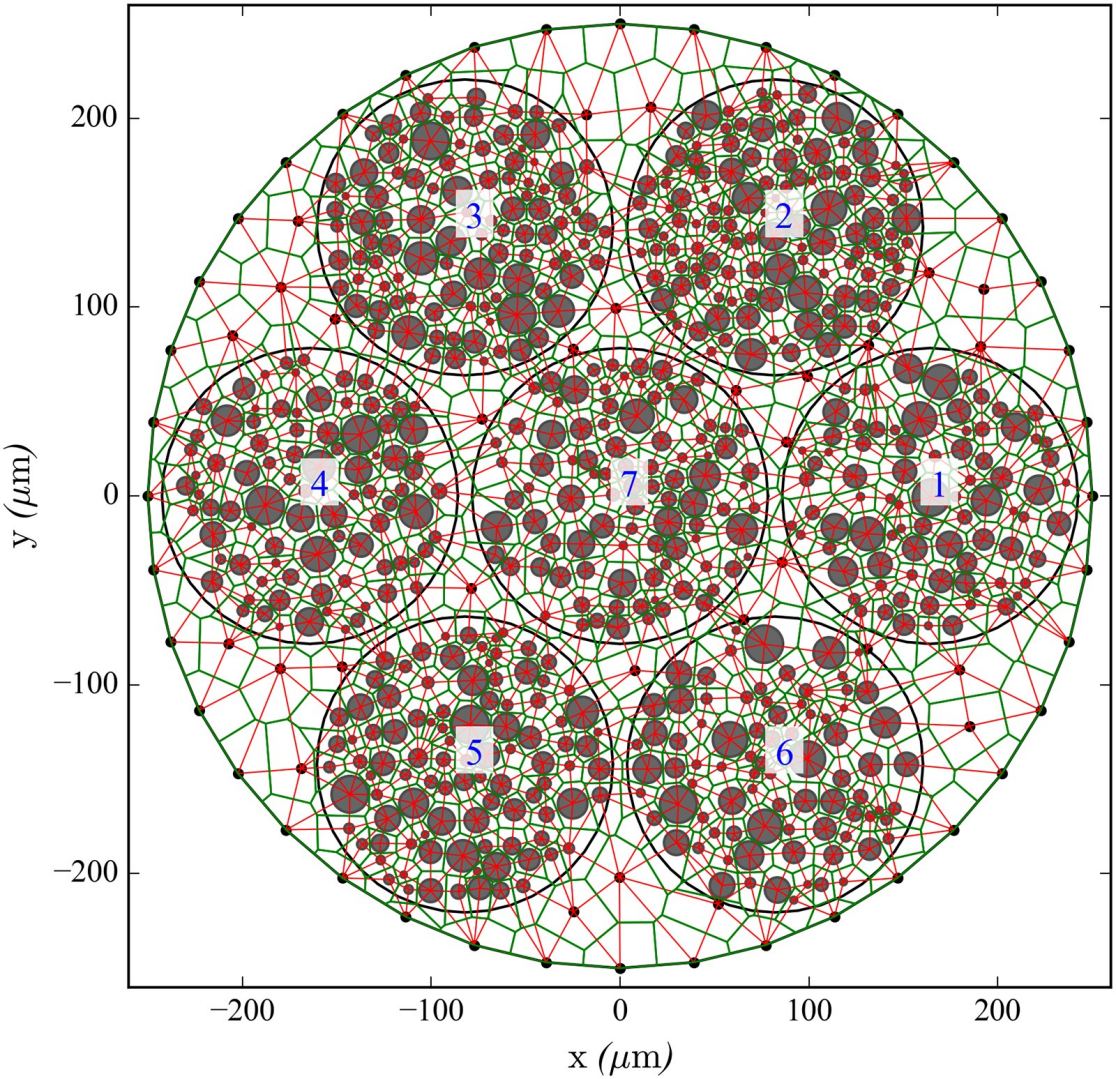
Power diagram and Delaunay triangulation of the nerve’s cross-section (Nerve 1). Discretisation of a nerve model’s cross-section Nerve 1 in polygons using a power diagram (green). Grey circles indicate the locations and diameters of the axons, which are embedded in seven fascicles (the blue labels number the fascicles). Black dots indicate points resulting from a Delaunay triangulation to discretise the epineurium, indicating the locations of NAELC. The dual Delaunay triangulation to the power diagram representing the connections with transverse resistors is represented with solid red thin segments. Note that while the nerve’s contour contains NAELC, the fascicles contours do not. This model is used in simulations in this work (see Nerve 1 in Fig 10 and Table 1).

**Fig 13:**
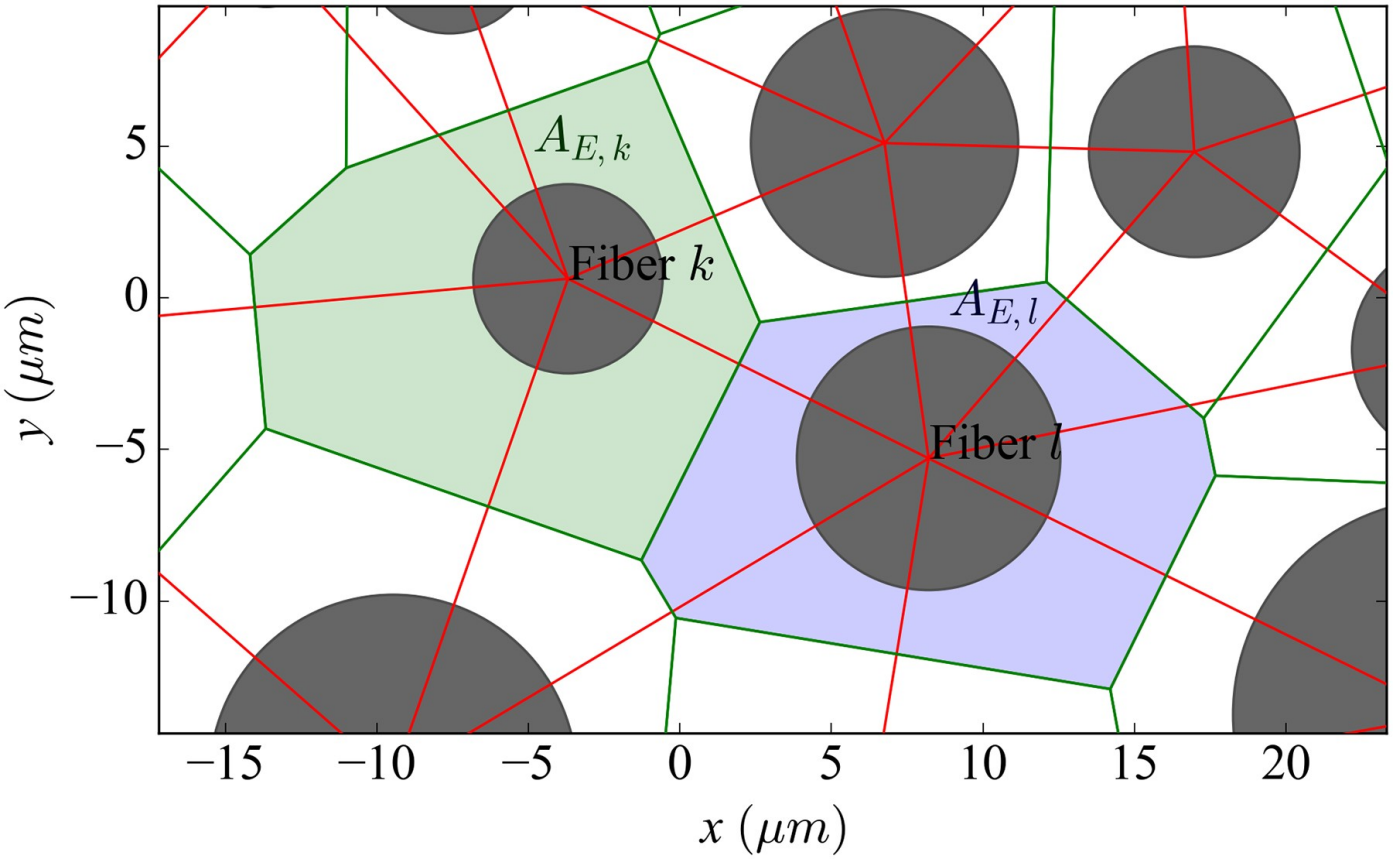
Power diagram and Delaunay triangulation of the nerve’s cross-section (zoomed). Cross-sectional view of a random fascicle including the tessellation (green lines) and the triangulation (red). Additional information is used to display the details of the connection between two randomly chosen nearest-neighbouring fibers *k* and *l*. The coloured areas represent the extracellular area assigned to the calculation of the longitudinal extracellular resistance of each fiber (green for fiber *k* and blue for fiber *l*).

The resistance per unit length of each longitudinal extracellular cable *r*_*L*,*k*_, equivalent to *R*_*L*_ in [43], is given by the extracellular cross-sectional area that can be assigned to each fiber. This represents the longitudinal resistance of the volume of extracellular medium surrounding each fiber. This extracellular cross-sectional area is given by the tessellation of the nerve described below (see also Figs 12 and 13).

#### Modelling the extracellular space for any number of axons and extrafascicular regions

Our model needs to extend this network to fit any number of randomly distributed axons across the fascicles. Also, the epineurium and regions of the nerve lying outside the fascicles that naturally do not contain axons may be large enough for us to need to model them with a finite-sized mesh with which to capture the electric field resulting from external stimulation with a certain level of detail.

The model creates an electrical resistive network among fibers by extending the nearest-neighbour connections model described above. In order to make the modelling of the extrafascicular regions compatible and consistent with this connectivity model, these regions were assigned longitudinal extracellular cables that were not directly attached to any fibers (referred to as non-axonal extracellular longitudinal cables, NAELC, from now on), but which were connected among them using the same method (with regular spacing among transverse resistors in this case). Therefore, the NAELC, all the extracellular cables directly attached to the fibers, and the transverse resistors, form the extracellular part of the RN.

The extension of this nearest-neighbour interaction model to the whole RN for an entire nerve requires a method to provide each axon and NAELC with a given extracellular area and to model the values for all the transverse resistors.

Such procedure is the following: A cross-sectional 2D slice of the epineurium is filled with points obtained from a Delaunay triangulation [44] of the nerve’s cross-sectional area. Each point falling outside the fascicles corresponds to the location of one NAELC, and any points falling inside the fascicles are removed. These points, together with the circles that define the locations and diameters of the axons, form a packing of non-intersecting circles in a two-dimensional space (the points may be regarded as zero-diameter circles for this purpose). This way, we obtain the set of positions for all fibers and NAELC (Fig 12).

A natural way to divide the space in an individual region for each circle of the packing is given by computing its power diagram, described as the Voronoi tessellation in the Laguerre geometry [45]. This tessellation technique divides the nerve’s cross-section into a set of convex polygons (Fig 12), each one containing one circle, thus existing a one-to-one correspondence between polygons and circles, and therefore assigning an extracellular cross-sectional area *A*_*E*,*k*_ to each fiber and NAELC.

Polygons containing the points on the nerve’s membrane are cropped so that they do not intersect the nerve’s outer space.

#### Longitudinal resistances of the resistor network

The longitudinal resistivity of an extracellular cable is determined using a lumped value of the longitudinal components of the resistivities of the tissues intersecting its polygon.

$$\rho_{k}^{Lu,L}=\sum_{i}a_{k,i}\cdot \rho_{i}^{L},$$ (5)

where *k* indicates the cable or polygon, *i* indicates the type of tissue and then, *a*_*k*,*i*_ is the cross-sectional area of tissue type *i* present in polygon *k* as a fraction of the total extracellular area enclosed by the polygon (this is, scaled over *A*_*E*,*k*_). In theory, in this study, this sum is made over two types of tissue: endoneurium and epineurium (*i* ∈ {*En*, *Ep*}). However, as mentioned above, we used the same value of $\rho_{i}^{L}$ for both. Nevertheless, this equation serves for any number of tissue types the modeller wishes to include.

The resistance per unit length of each extracellular cable is:

$$r_{L,k}=\frac{\rho_{k}^{Lu,L}}{A_{E,k}},$$ (6)

where *A*_*E*,*k*_ is the aforementioned extracellular cross-sectional area of the polygon. If *A*_*P*,*k*_ is the total area of the polygon and *D*_*k*_ is the diameter of fiber *k*, *A*_*E*,*k*_ is given by:

$$A_{E,k}=A_{P,k}-\pi D_{k}^{2}$$ (7)

If the polygon does not contain a fiber but a NAELC,

$$A_{E,k}=A_{P,k}$$ (8)

#### Transverse resistances of the resistor network

Sides shared by adjacent polygons in the power diagram represent electrical contacts between the polygons (which is equivalent to surface contacts between polygonal prisms because the polygons are extruded along the *z*-axis) and determine which cables or fibers are coupled by transverse resistors. The weighted Delaunay triangulation dual to the power diagram [45] (red lines in Figs 12 and 13) indicates these connections. The resistance of such a contact depends directly on the distance *d*_*C*,(*k*,*l*)_ between the centers of the two circles and inversely on the product of its segment’s length *s*_*k*,*l*_ (green segment joining the two coloured polygons in Fig 13) times its length along the *z*-axis $c_{k,l}^{n}$. Hence the values of the extracellular transverse resistors between two fibers are:

$$R_{T,(k,l)}^{n}=\rho_{k,l}^{Lu,T}\frac{d_{C,(k,l)}}{c_{k,l}^{n}\cdot s_{k,l}},$$ (9)

The transverse component of the lumped resistivity $\rho_{k,l}^{Lu,T}$ is computed in the following way:

$$\rho_{k,l}^{Lu,T}=\sum_{i}b_{k,l}^{i}\cdot \rho_{i}^{T},$$ (10)

where $b_{k,l}^{i}$ is the distance crossed by the resistor within the tissue of type *i*, scaled over *d*_*C*,(*k*,*l*)_.

For merely geometrical arrangements, the perineurium is modelled as an infinitely thin layer, so it does not affect the calculations of *r*_*L*_. Yet its nominal thickness was not ignored for the calculations of the resistances of transverse resistors crossing it, since its thickness is known to affect the results of stimulation [42]. Its thickness was added in the calculation of the corresponding $R_{T,(k,l)}^{n}$ in the following way:

$$R_{T,(k,l)}^{n}=\frac{1}{c_{k,l}^{n}\cdot s_{k,l}}(\rho_{k,l}^{Lu,T}(d_{C,(k,l)}-n_{P}\Delta_{P})+\rho_{P}^{T}n_{P}\Delta_{P}),$$ (11)

where *n*_*P*_ is the number of perineurial membranes crossed by a resistor (1 between an axon and a NAELC, 2 between two axons in different fascicles, 0 otherwise).

NAELC are always discretised in regular intervals, using the shortest internodal length in the nerve for $c_{k,l}^{n}$. Transverse resistors connecting a NAELC and a fiber are located on the nodes of Ranvier of the fiber.

### Nerve’s external environment and electrodes

The nerve was centered along the axis of a larger cylindrical container (*z*-axis) filled with a saline bath. The surface of this larger cylinder was connected to ground (zero potential), as done before by [12]. For modelling purposes, this can be used as a sufficient representation of the animal’s body surrounding the nerve, assuming that in a real experiment, the ground would presumably be found on a distant location, right outside the animal’s body or in the Central Nervous System.

This model framework allows the user to define cuff electrodes for stimulation. We used cuff electrode models based on [46]. These electrodes are 4.25 mm long and contain four rings separated by 750 *μ*m each. Each ring contains four pads, placed at 0°, 90°, 180° and 270° with respect to the *x*-axis. More details about the geometry and materials of these electrodes can be found in [46]. In this work, the cuff model was simplified by leaving only one ring in the center, and by adapting the inner diameter to the nerve model diameter. Stimulation from the pads is simulated using current point sources on the nerve’s membrane’s NAELC in contact with the desired pads.

The current path between the points on the nerve’s membrane and the container’s walls was assumed to be purely radial (hence no longitudinal currents are allowed across the bath or the cuff insulators). For this, all points in the discretised nerve lying on its membrane (which are given by the triangulation hull in the cross-section) were connected to the container’s cylindrical wall using radially aligned resistors. The resistance per unit length for each of these resistors was estimated from the geometry of the bath (see Tables 2 and 3 for variables and parameters):

$$R_{G}=\frac{\rho_{I}\Delta_{I}+\rho_{S}\Delta_{S}}{\left(\pi D_{N}/n_{H}\right)},$$ (12)

where:

$$\Delta_{I}+\Delta_{S}=\Delta_{C}$$ (13)

In the regions of the nerve (along its length) not covered by the cuffs, the membrane was directly in touch with the saline bath and Eq 12 then becomes:

$$R_{G}=\frac{\rho_{S}\Delta_{C}}{\left(\pi D_{N}/n_{H}\right)}$$ (14)

All NAELC and extracellular cables of fibers were connected to ground on both ends since they are assumed to be in contact with the container’s bases. The ends of the intracellular domains of the fibers, however, are treated as sealed ends and do not have such connections.
